# Biosynthesis and Characterization of Zinc Oxide Nanoparticles and Their Impact on the Composition of Gut Microbiota in Healthy and Attention-Deficit Hyperactivity Disorder Children

**DOI:** 10.3389/fmicb.2021.700707

**Published:** 2021-08-05

**Authors:** Guoling Zhou, Rongrong Yu, Temoor Ahmed, Hubiao Jiang, Muchen Zhang, Luqiong Lv, Fahad A. Alhumaydhi, Khaled S. Allemailem, Bin Li

**Affiliations:** ^1^Hangzhou Seventh People’s Hospital (HSPH), Hangzhou, China; ^2^School of Education Science and Technology, Zhejiang University of Technology, Hangzhou, China; ^3^Institute of Biotechnology, Zhejiang University, Hangzhou, China; ^4^Department of Medical Laboratories, College of Applied Medical Sciences, Qassim University, Buraydah, Saudi Arabia

**Keywords:** attention-deficit hyperactivity disorder, amplicon sequencing, bacterial community, gut bacteria, zinc oxide nanoparticles

## Abstract

Attention-deficit hyperactivity disorder (ADHD) seriously affects children’s health, and the gut microbiome has been widely hypothesized to play a role in the regulation of ADHD behavior. The present study aims to the biosynthesize of zinc oxide nanoparticles (ZnONPs) by using *Acinetobacter johnsonii* strain RTN1, followed by their characterization through state-of-the-art material characterization techniques, viz., UV–vis spectroscopy, Fourier transform infrared spectroscopy, and transmission and scanning electron microscopic analyses with energy dispersive spectrometry. Moreover, we investigated and compared the population composition of gut microbiota and their susceptibility to biogenic ZnONPs between healthy and ADHD children based on the traditional plate method and 16S rRNA amplicon sequence analysis. The antibacterial effect of ZnONPs against gut bacteria was also determined by measurement of live cell number, living/dead bacterial staining test, and flow cytometry observation. The present study revealed that the number of live gut bacteria in healthy children was more than 10-fold higher than that in ADHD children; however, the community structure of gut bacteria has changed, while greater diversity was found in gut bacteria from ADHD children. In addition, we found that the number of live gut bacteria in healthy and ADHD children was reduced by ZnONPs, which shows an increased and reduced effect in composition of gut bacteria from healthy and ADHD children, respectively. It was also noted that the main mechanism of ZnONPs may be to inhibit the growth of gut bacteria rather than to kill them, while the nanoparticle-resistant strains in healthy children is also different from that in ADHD children. Some representative bacteria, in particular nanoparticle-resistant bacteria, were successfully isolated and identified. Overall, this study revealed the potential correlation of ADHD with gut bacteria and provided a new possibility to prevent ADHD by the combination of nanoparticle and its resistant bacteria.

## Introduction

Attention-deficit hyperactivity disorder (ADHD) is one of the most prominent developmental and neuropsychiatric disorders characterized by problems with inattention, impulsivity, and hyperactivity, which have been increasingly diagnosed in the last decade and affects approximately 8–12% of school-aged children worldwide (Luo et al., 2019). Children with ADHD are extremely active and unable to concentrate on anything for very long, with the result that they find it difficult to learn and often behave in inappropriate ways (Wang et al., 2020). Although pharmacological treatments can reduce symptoms, they are often unsatisfactory due to side effects, fail to prevent or alter long-term course, and are discontinued due to patient and family preferences (Hiergeist et al., 2020). Several studies have reported that the incidence of ADHD may have a certain degree of heritability, and the abnormally expressed genes in children with ADHD genes are associated with norepinephrine and serotonin [5-hydroxytryptamine (5-HT)] as well as dopamine synthesis and transmission (Karmakar et al., 2017), Furthermore, the mutual effects of environmental factors and genetic polymorphism are also attracting much attention in ADHD studies (Hayashi and Iwanami, 2018). Although various theories have been proposed, the pathogenic mechanisms underlying ADHD have not been fully clarified.

Interestingly, there is also growing evidence supporting the role of the human gut microbiome in the development of ADHD (Sampson and Mazmanian, 2015; Boonchooduang et al., 2020). It has been well known that the gut microbiota is the largest ecosystem in the human body, which is believed to play a pivotal role in human health and disease such as psychiatric disorders in multiple ways (Martins-Silva et al., 2021). In practicality, the brain–gut axis theory proposes that the gut microbiota can influence brain function and behaviors through involvement in physiological homoeostasis, immunological development, glutathione metabolism, amino acid metabolism, etc (De Vadder et al., 2014). Therefore, it is necessary for us to investigate the change in the composition of gut microbiome from ADHD children, which will provide a new insight to elucidate the mystery of this complex disease.

In recent years, green synthesized nanomaterials have gained much attention because of their potential use as therapeutic agents (Kummara et al., 2016; Alphandéry, 2020). Although many physical and chemical approaches have been used to produce nanomaterials, the microbe-mediated synthesis of zinc oxide nanoparticles (ZnONPs) is more favorable than traditional methods because of their unique physicochemical properties such as crystalline structure, surface area, size, and shape, and more stable nature (Ahmed et al., 2021c). Recently, biologically synthesized nanoparticles (NPs) have been well documented and served as the best alternative to previously available expensive and environment-damaging physicochemical technologies (Ahmed et al., 2020, 2021a). Indeed, our previous studies have indicated that green synthesized metallic NPs have potential bacteriodical effect against human pathogenic Gram-positive bacteria *Staphylococcus aureus* and gram-negative bacteria *Escherichia coli* (Ahmed et al., 2020). Although the evaluation of microbiota/microbiome-mediated effects of nanomaterials is in its early infancy, few experimental studies have been carried out on the interaction between nanomaterials and gut microbiome/microbiota, which revealed a double-face effect in the relation between nanomaterials and human health with possible adverse effects from one side and with possible beneficial application on the other (Pietroiusti et al., 2016). These differential results may be mainly attributed to the difference in the type, time, and dose of the used NPs. In previous studies, biosynthesized ZnONPs are described as being biocompatible and as not inducing cytotoxicity toward healthy cells (Agarwal et al., 2018; Saravanan et al., 2018; Suresh et al., 2018).

The aim of this study was to investigate the population number and composition of gut microbiota from healthy and ADHD children and their susceptibility to biogenic ZnONPs based on the analysis of 16S rRNA amplicon sequencing in a combination of the plate counting method, living/dead bacteria staining test, flow cytometry observation, and the16S rDNA sequence analysis. The result of this study will provide a new insight for us to prevent ADHD by the combined use of NP its resistant bacteria.

## Materials and Methods

### Biosynthesis and Characterization of ZnONPs

Fresh culture of *Acinetobacter johnsonii* strain RTN1 (accession no. MT173803) was collected from the culture collection of the Institute of Biotechnology, Zhejiang University, China. The ZnONPs were synthesized by using bacterial strain RTN1, according to Selvarajan and Mohanasrinivasan (2013). In brief, the bacterial strain RTN1 was grown overnight in nutrient broth (NB) media at 28 ± 2°C in a shaking incubator, and supernatant of bacterial culture was collected after centrifugation at 5,000 *g* for 10 min. For the biosynthesis of ZnONPs, a ratio of 1:1 of 0.1 M ZnSO_4_·7H_2_O and the culture supernatant of strain RTN1 were mixed in a 250-ml Erlenmeyer flask, and the reaction mixture was subjected to heating on a water bath up to 80°C for 15 min. The visible white precipitates that appeared at the bottom of the flask confirmed the synthesis of ZnONPs. Afterward, ZnONPs were collected by centrifugation at 10,000 *g* for 10 min and washed three times with distilled water.

The synthesis of ZnONPs in a reaction mixture was observed using a UV–vis spectrophotometer (Shimadzu Corporation, Kyoto, Japan) at 300- to 700-nm wavelength range (Ahmed et al., 2021c). The Fourier transform infrared spectroscopy (FTIR) analysis was carried out to identify different functional groups present in the ZnONPs by using a FTIR spectrometer (Vector 22; Bruker, Germany) in the spectral region range of 4,000–500 cm^−1^. Moreover, the size, shape, and element composition of biogenic ZnONPs were observed using scanning electron microscopy (SEM; SU8010, Hitachi, Japan) and transmission electron microscopy (TEM; JEM-1230, JEOL, Akishima, Japan) analyses. Moreover, the elemental composition of ZnONPs was confirmed through energy-dispersive spectroscopy (EDS; Oxford, United Kingdom) at 20 keV, according to Ahmed et al. (2021b).

### Collection of Fecal Samples

In this study, we recruited a total of 44 children with ADHD (mean age: 6.9 years) who were treated in the Pediatric Outpatient Department of Hangzhou Seventh People’s Hospital from January 2019 to July 2020. In addition, a total of 38 healthy children were recruited from primary schools in Hangzhou in this study as the control (mean age: 8.6 years) for analysis of fecal microbiota. All children were between 6 and 12 years old, while the identities of the ADHD children were determined in Hangzhou Seventh People’s Hospital based on the current criteria of the DSM-5 (“Diagnosis and Statistical Mannual of Mental Disorders, Fifth Edition”). After collecting the feces from each recruited children in the corresponding centrifuge tube and then putting each sample in an ice bag, all fecal samples were stored at −20°C for further use.

### Preparation of the Mixed Fecal Samples

The mixed fecal samples of ADHD children were prepared by taking about 1.0 g of fresh fecal samples from each ADHD children and then dissolving them into 10 ml of sterile distilled water. Following the thorough shaking and mixing and centrifugation at 10,000 *g* for 3 min at room temperature, the pellets were collected and stored at 4°C for further use. A similar procedure was applied for the preparation of the mixed fecal samples of healthy children.

### Susceptibility of Gut Microbiota to ZnONPs

The suspension of gut microbiota was prepared by dissolving the mixed fecal samples from ADHD and healthy children in distilled water. Susceptibility of gut microbiota to ZnONPs was determined by adding the stock solution (1.0 mg/ml) of ZnONPs into the suspension of the mixed fecal samples to obtain a final concentration of 20 μg/ml.

### Counting Surviving Bacterial Cells

Suspensions of gut bacteria were prepared by dissolving the mixed fecal samples from ADHD and healthy children in distilled water and then filtering with four layers of gauze to remove impurities. The surviving bacterial cells were counted as described by Li et al. (2008). In brief, gut bacterial suspensions were 10-fold serially diluted, and 10-μl samples were inoculated on nutrient agar medium in sextuplicate for each dilution and were incubated for 48 h at 37°C. After incubation, the surviving cells on the agar were counted based on the colony-forming units, and then the mean value of the cells at the lowest dilution was calculated. This experiment was repeated twice with six replicates of each treatment.

### Live/Dead Cell Staining

To observe the damage and intact membranes in cells of gut bacteria exposed to 20 μg/ml ZnONPs, we performed an analysis of live/dead cell staining. In brief, the mixed fecal samples from ADHD and healthy children were dissolved in distilled water and then filtered with four layers of gauze to remove impurities. Live/dead staining of gut bacteria was carried out as described by Yu et al. (2020) according to the protocol of BacLight bacterial viability kit (Invitrogen) following the induction of isopropyl-beta-d-thiogalactopyranoside (IPTG) with the final concentration of 1 mM. There are two nucleic acid stains in the kit named (i) red fluorescent (propidium iodide stain) for dead bacteria and (ii) green fluorescent (SYTO 9 stain) for live bacteria. Fluorescence in the sample was detected using the Olympus inverted confocal microscope.

### Flow Cytometry Observation

Flow cytometry was detected as described by Ogunyemi et al. (2019b) with minor revision. In brief, after IPTG was induced for 12 and 24 h, the harvested bacteria were centrifuged at 5,000 *g* for 10 min, then washed three times using ddH_2_O, and, finally, the PI solution was diluted in bacterial suspension to 50 mg/L, and the bacteria were stained for 20 min in the dark and then washed. The staining cells can be monitored using flow cytometry (Beckman Coulter, Gallios, Germany).

### 16S rRNA Amplicon Sequencing Analysis

#### Extraction of Genomic DNAs

Total DNA was extracted from stool samples using the OMEGA Soil DNA Kit (D5625-01; OMEGA Bio-Tek, Norcross, GA, United States) according to the manufacturer’s recommendations for human stool samples. The quantity and quality of the extracted DNA were detected by the NanoDrop ND-1000 spectrophotometer (Thermo Fisher Scientific, Waltham, MA, United States) and agarose gel electrophoresis, respectively. Extracted DNA was stored at −20°C until PCR amplifcation and Illumina MiSeq DNA sequencing.

#### 16S rRNA Amplicon Sequencing

The DNA samples were subjected to the first-run PCR, where the specified forward primer 338F (5ꞌ-ACTCCTACGGGAGCAGCA-3ꞌ) and reverse primer 806R (5ꞌ-GGACTACHVGGGTWTCTAAT-3ꞌ) were designed to amplify the V3–V4 genomics region of bacterial 16S rRNA genes. The PCR system contain 5 μl of buffer solution (5×), 0.25 μl of fast pfu DNA polymerase (5 U/μl), 2 μl of dNTPs (2.5 mM), 1 μl of forward and reverse primers (10 μM), 1 μl of DNA template solution, and 14.75 μl of ddH_2_O. The thermal cycle consists of an initial denaturation of 5 min at 98°C, followed by 25 cycles of denaturation at 98°C for 30 s, annealing at 53°C for 30 s, expansion at 72°C for 45 s, and, finally, an extension of 5 min at 72°C. The PCR products were purified by using V azyme VAHTSTM DNA Clean Beads (V azyme, Nanjing, China) and quantified by Quant-iT PicoGreen dsDNA detection kits (Invitrogen, Carlsbad, CA, United States). Following the confirmation with gel electrophoresis and polymerization in equal quantities, the PCR-amplified products were then subjected to library preparation for 16S rRNA sequencing using the 2 × 250-bp paired-end protocol. We prepared a DNA library according to the 16S rRNA Sequencing Library Preparation instructions (Illumina, CA, United States). The prepared amplicons were sequenced on the MiSeq platform (Illumina, CA, United States) with the MiSeq Reagent Kit v3 (600 cycles) from Shanghai Personal Biotechnology Co. Ltd. (Shanghai, China).

#### Bioinformatics Analysis

Sequence data analysis was carried out as described by Stevens et al. (2019) with minor modification using QIIME2 (version 2020.06) and R package (v3.6.0). In brief, 16S rRNA gene amplicon sequences were demultiplexed and preprocessed. Barcode and primer removal, quality control, amplicon sequence data correction, phiX filtering, and dereplication were verified using the DADA2 sofware package43 (Callahan et al., 2016). A phylogenetic tree was constructed with fasttree2. The structural changes of the microbial community in different samples were investigated by calculating the alpha diversity index, which was determined by using Shannon, Simpson, Pielou’s evenness, beta diversity index including weighted and unweighted UniFrac, Jaccard distance, and Bray–Curtis. Comparing the richness and evenness of amplicon sequence variant (ASV) among samples, each sample is limited to the same number of reads (33,680 sequences, the smallest number of sequences). Principal components analysis (PCA) was performed on the phylum, genus, and species levels of each sample.

### Statistical Analysis

The software STATGRAPHICS Plus, version 4.0 (Copyright Manugistics Inc., Rockville, MD, United States) was used to perform the statistical analyses. Levels of significance (*p* < 0.05) of main treatments and their interactions were calculated by the analysis of variance (ANOVA) after testing for normality and variance homogeneity.

## Results and Discussion

### Biosynthesis and Characterization of ZnONPs

**Figure 1:**
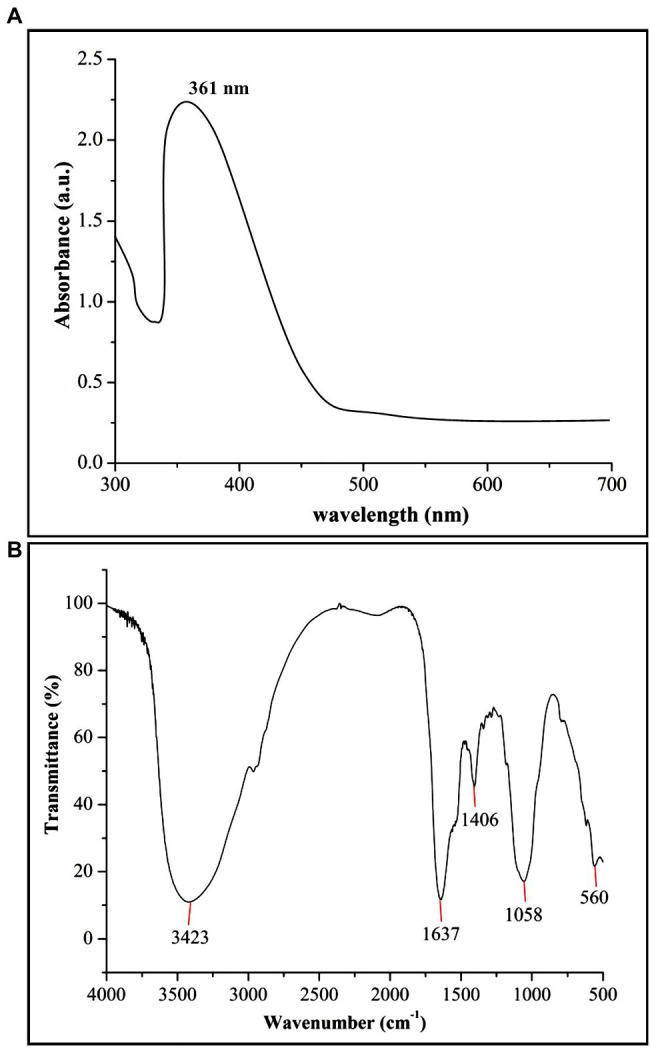
Characterization of biogenic zinc oxide nanoparticles (ZnONPs) using **(A)** UV–vis spectroscopy and **(B)** Fourier transform infrared spectroscopy (FTIR) analysis.

In the present study, biogenic ZnONPs were extracellularly synthesized by using a bacterial strain RTN1, and the maximum precipitate clustered at the bottom of the flask was determined at 0.1 M ZnSO_4_·7H_2_O concentration. Similarly, Ahmed et al. (2021c) observed the extracellular biosynthesis of ZnONPs from *Bacillus cereus* as a capping and reducing agent. Several studies reported that the microbe-mediated synthesis of NPs are eco-friendly, less toxic, and more stable as compared with chemical synthesis (Gericke and Pinches, 2006; Ali et al., 2020). The production of ZnONPs was further confirmed by the UV–vis absorption peak measured at 361 nm (Figure 1A), which is in agreement with the study of Malaikozhundan et al. (2017), who synthesized the *Bacillus thuringiensis*-coated ZnONPs and observed the UV–vis spectral peak at 373 nm, which is consistent with our study.

**Figure 2:**
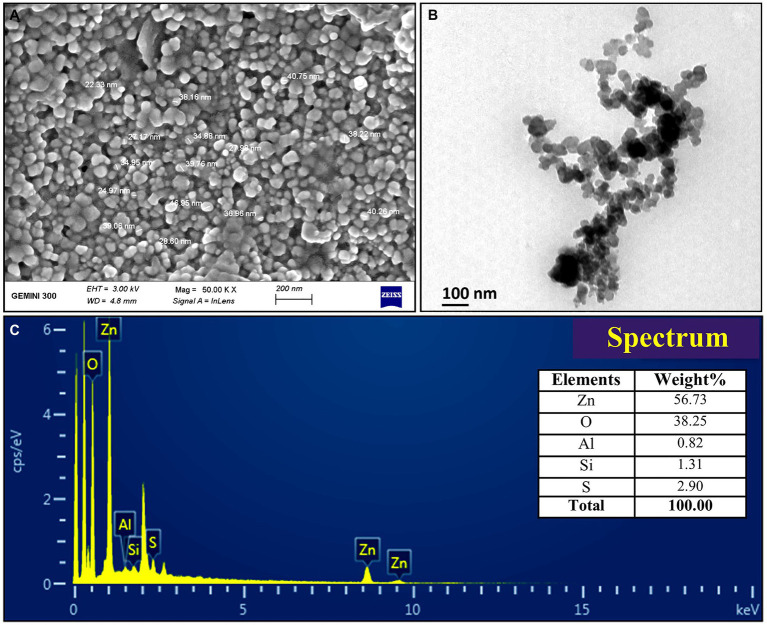
Characterization of biogenic ZnONPs using **(A)** scanning electron microscopy (SEM), **(B)** transmission electron microscopy (TEM), and **(C)** EDX spectrum.

The FTIR analysis was used to confirm the presence of different functional groups involved in the long-term stabilization of biogenic ZnONPs. The FTIR spectra of ZnONPs showed the absorption peaks at 3,423, 1,637, 1,406, and 1,058 cm^−1^ and a weak peak at 560 cm^−1^ (Figure 1B). The strong peaks at 3,423 and 1,637 cm^−1^ were revealed in the presence of the hydroxyl group (–OH) of alcohol and C=C stretching of alkene, respectively. The peaks at 1,406 and 1,058 cm^−1^ were attributed to the stretching of O–H bending and C–O stretching of the alcohol group, respectively. Our FTIR results are consistent with the findings of Selvarajan and Mohanasrinivasan (2013), who biologically produced the ZnONPs from the *Lactobacillus plantarum* VITES07. Overall, it can be concluded that these functional groups as capping agents might act as a barrier to prevent agglomeration among ZnONPs and maintain their long-term stability. Moreover, the SEM and TEM analyses revealed that biogenic ZnONPs have spherical shapes with particle size ranging from 22 to 46 nm (Figure 2). The results of the EDS spectral analysis confirmed the presence of zinc (56.73%), oxygen (38.25%), aluminum (0.82%), sulfur (2.90%), and silicon (1.31%) in biogenic ZnONPs (Figure 2C), which is consistent with the previous studies (Ogunyemi et al., 2019a, 2020).

### Changes of Gut Bacteria by Counting the Surviving Cells

The latest research in childhood ADHD has slowly shifted from just concerned about genetics to more and more focus on the gut bacteria, which play an important role (Bundgaard-Nielsen et al., 2020; Checa-Ros et al., 2021). Indeed, changes in gut microbiota profiles in children with ADHD have been reported in several studies (Hiergeist et al., 2020). In order to assess the possible role of the gut microbiota in disease development and to open new avenues for ADHD treatment, we conducted a case–control study to investigate and compare the number and composition of gut bacteria from the fecal samples of healthy and ADHD children based on the traditional plate method and a 16S rRNA amplicon sequencing approach. In addition, we investigated the antibacterial effect of ZnONPs against gut bacteria by measurement of live cell number, living/dead bacterial staining test, and flow cytometry observation, while the change in composition of gut microbiome by ZnONPs was determined by sequencing of 16S rRNA gene from the obtained resistant strains and 16S rRNA amplicon sequencing of fecal samples from healthy and ADHD children. Overall, the results revealed the importance of gut bacteria in ADHD and gave a new strategy to prevent ADHD by the combination of NP and its resistant bacteria.

**Figure 3:**
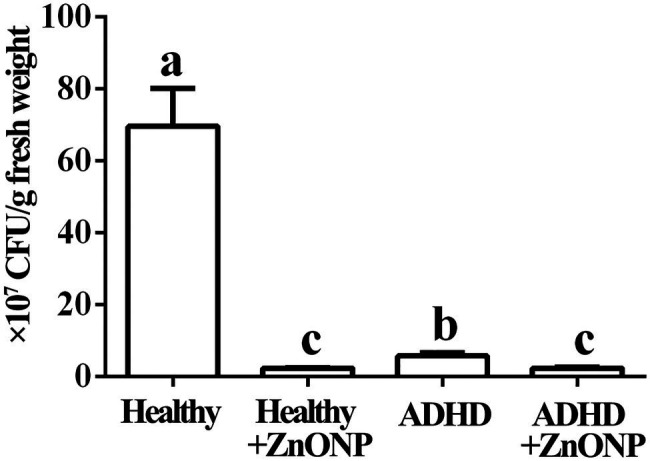
Numbers of gut bacteria in fecal samples from the healthy and attention-deficit hyperactivity disorder (ADHD) children by the plate counting method. Vertical bars represent standard errors of the means (*n* = 6). Bars followed by the same letter(s) are not significantly different (*p* ≤ 0.05).

The results from this study indicated that in the absence of ZnONP, the number of the live bacteria is 6.95 × 10^8^ CFU/g fresh feces from healthy children, while there was a 91.75% reduction in the number of the live bacteria in fresh feces from children with ADHD compared to the healthy children. Furthermore, the addition of ZnONP with a final concentration of 20 μg/ml caused a 96.69% reduction in the number of live bacteria in fresh feces from healthy children. In comparison, the addition of ZnONP with a final concentration of 20 μg/ml caused a 96.73% reduction in the number of live bacteria in fresh feces from children with ADHD compared to the healthy children (Figure 3). The result of this study indicated that ADHD caused a significant reduction in the number of live gut bacteria, which is consistent with the result of several previous studies (Stevens et al., 2019; Szopinska-Tokov et al., 2020; Wan et al., 2020). Furthermore, our results also indicated that the number of live gut bacteria was significantly reduced by NPs regardless of healthy or ADHD children. In agreement with the data of this study, Abid et al. (2020) reported that the biosynthesized iron oxide NPs had strong antibacterial activity against the gram-negative bacteria *Klebsiella pneumonia* and Gram-positive bacteria *S. aureus*. However, more reduction was observed in healthy children compared to that of those with ADHD. To the best of our knowledge, this is the first report about the effect of NPs on the number of gut bacteria from healthy and ADHD children by incubating fecal sample on the NA medium.

### Changes in Number of Gut Bacteria by Live-Dead Staining

**Figure 4:**
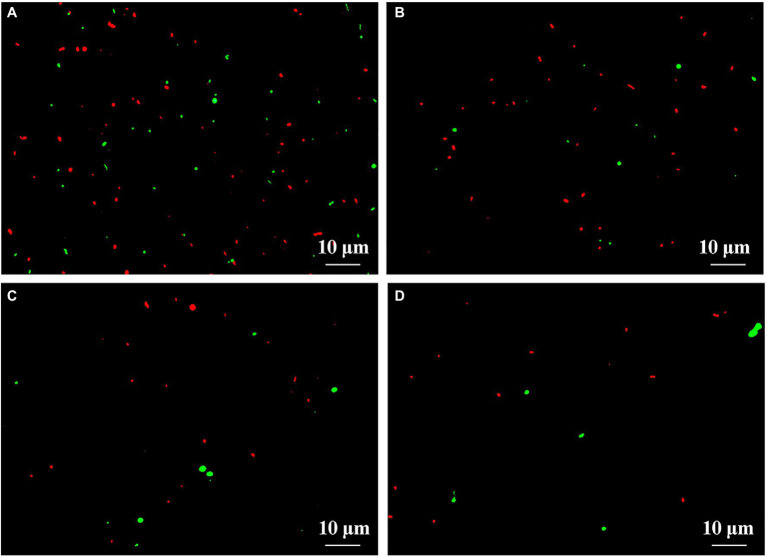
Effect of ZnONP on number of gut bacteria from healthy and ADHD children by live-dead staining. **(A)** Healthy; **(B)** healthy + ZnONP; **(C)** ADHD; and **(D)** ADHD + ZnONP.

This result from microscopic observation indicated a lot of live (fluoresce green) and dead (fluoresce red) bacteria in fresh feces from healthy children in the absence of ZnONP. However, the addition of ZnONP at the concentration of 20 μg/ml caused a marked reduction in the cell numbers of live bacteria in fresh feces from healthy children. In addition, compared to the healthy children, the cell number of live and dead gut bacteria in fresh feces from ADHD children was markedly reduced regardless of the presence or absence of ZnONP (Figure 4). This result from staining of the live/dead bacterial cells indicates that the ZnONP has a greater inhibition in the growth of gut bacteria in healthy children compared to that of ADHD children, which is consistent with the result of a bacterial number using the plate counting method. However, no more dead cells were observed in the presence of ZnONP regardless of healthy or ADHD children, this indicates that the reduction in the number of live cells by this NP may be mainly attributed to its strong inhibition in bacterial replication but not killing bacteria. In contrast with the result of our study, several of our previous studies have revealed that NPs have a bacteriostatic rather than bactericide effect on various bacteria (Masum et al., 2019; Ahmed et al., 2021a).

### Changes in Number of Gut Bacteria by Flow Cytometry Observation

**Figure 5:**
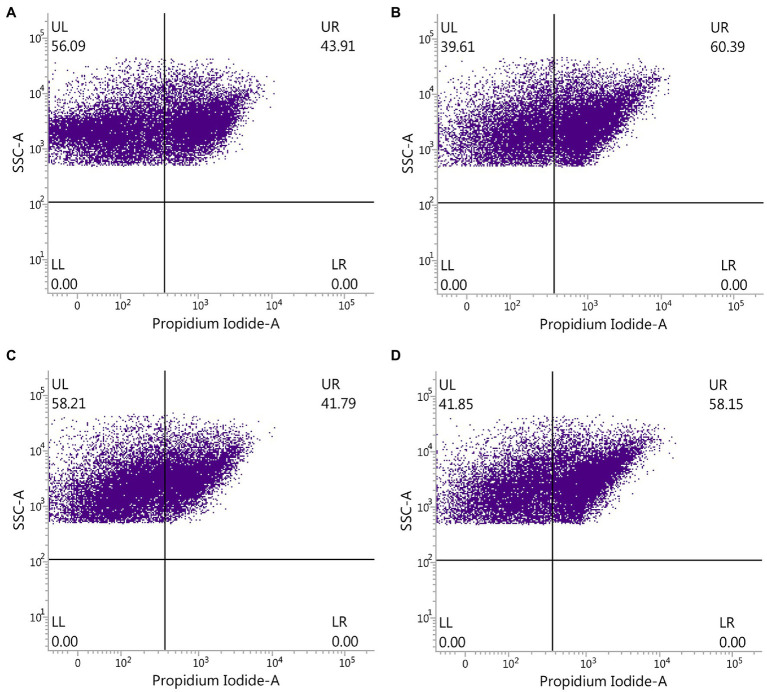
Effect of ZnONP on number of gut bacteria from healthy and ADHD children by flow cytometry observation. **(A)** Healthy; **(B)** healthy + ZnONP; **(C)** ADHD; and **(D)** ADHD + ZnONP.

The effect of ADHD and ZnONP on gut bacteria was further determined by comparing the ratio of live with dead gut bacteria in fresh feces from healthy and ADHD children in the presence or absence of ZnONP calculated by using flow cytometry observation. The results of this study indicated that the percentage of live and dead gut bacteria in healthy children was 56.09 and 43.91%, respectively, while the percentage of live and dead gut bacteria in healthy children was 39.61 and 60.39%, respectively, in the presence of ZnONP. Furthermore, the percentage of live and dead gut bacteria in ADHD children was 58.21 and 41.79%, respectively. In comparison, the percentage of live and dead gut bacteria in ADHD children was 41.85 and 58.15%, respectively, in the presence of ZnONP (Figure 5). This study indicated a similar ratio of live to dead gut bacteria in fresh feces between healthy and ADHD children in the absence of ZnONP. However, a greater number of live bacteria was observed in the former compared to the latter. However, the percentage of dead gut bacteria was greatly increased by ZnONP regardless of healthy or ADHA children. Indeed, the addition of ZnONP caused a 37.53% increase in the ratio of dead gut bacteria in feces from healthy children, while the addition of ZnONP caused a 39.15% increase in the percentage of deadly gut bacteria in the feces from ADHD children. This result indicated that the antibacterial activity of ZnONP may be partially attributed to its bactericidal ability, which is a little different from the result of the live/dead bacterial cell staining, indicating that the former may have a higher resolution in the antibacterial activity than the latter. Interestingly, these results suggested that the reduction in number of gut bacteria is mainly due to ADHD other than ZnONP, which caused a similar increase between healthy and ADHD children in the percentage of dead cells.

### Identification of Gut Bacteria Resistant to NPs

Several researchers have tried to investigate the population of the general gut microbiota in ADHD children using the nonculture method (Stevens et al., 2019; Wang et al., 2020), which gives a comprehensive analysis in the composition of gut bacteria from ADHD children. Indeed, the nonculture method has the advantage of quicker results, less sample, and less cost, but it also has the disadvantage of not being able to know whether these gut bacteria is live or dead. In contrast, the culture method in this study provides an alternate strategy to the analysis of the gut microbiota profiles by isolating the gut bacteria and then focusing on the live bacteria in children with ADHD. In order to clarify the main bacterial species that resistant to NPs, the main representative bacterial colonies. The collected colonies from the healthy children were generally classified into four different groups, while the colonies collected from the ADHD children were classified into three different groups. The identity of each group was further determined based on the analysis of 16S rDNA sequence of the representative isolates, which were collected by picking out from the NA medium supplement with ZnONP of 20 μg/ml based on the shape, color, and edge of colony. Indeed, sequence analysis of representative colonies (strains 2-1, 2-2, 2-3, and 2-4) indicated that *Escherichia fergusonii*, *B. cereus*, *E. coli*, and *Escherichia* sp. were the main bacterial species in fresh feces from healthy children, while sequence analysis of representative colonies (strains 4-1, 4-2, and 4-3) indicated that *B. cereus*, *Bacillus paramycoides*, and *Bacillus anthracis* were the main bacterial species in fresh feces from ADHD children (Table 1). Results of this study indicated that the main gut bacterial community resistant to NPs in fecal samples from healthy children was greatly different from that from ADHD children. The main NP-resistant gut bacteria in healthy children belongs to the genus *Escherichia*, while the main NP-resistant gut bacteria in healthy children belongs to the genus *Bacillus*. This may be mainly due to the difference in community structure between healthy and ADHD children. In agreement with the result of our study, the change of community structure in ADHD children has been reported in many previous studies (Stevens et al., 2019; Wang et al., 2020). Interestingly, the result clearly indicated that some gut bacteria show great resistance to NPs, which may be used to adjust the community structure of gut bacteria by combining NPs.

**Table 1:** Sequence similarity of the main representative strains from the fecal samples of the healthy and ADHD children.

Bacterial strains	Sources	Bacterial identity (sequence similarity of 16S rDNA)	Accession no.
Strain 2-1	Healthy + ZnONP	*Escherichia fergusonii* (99.79%)	MW664943
Strain 2-2	Healthy + ZnONP	*Bacillus cereus* (99.12%)	MW664944
Strain 2-3	Healthy + ZnONP	*Escherichia coli* (99.58%)	MW664945
Strain 2-4	Healthy + ZnONP	*Escherichia* sp. (99.38%)	MW664946
Strain 4-1	ADHD + ZnONP	*Bacillus thuringiensis* (99.06%)	MW664947
Strain 4-2	ADHD + ZnONP	*Bacillus paramycoides* (98.80%)	MW664948
Strain 4-3	ADHD + ZnONP	*Bacillus anthracis* (99.69%)	MW664949

### Community Diversity Determined by 16S rRNA Amplicon Sequencing

**Figure 6:**
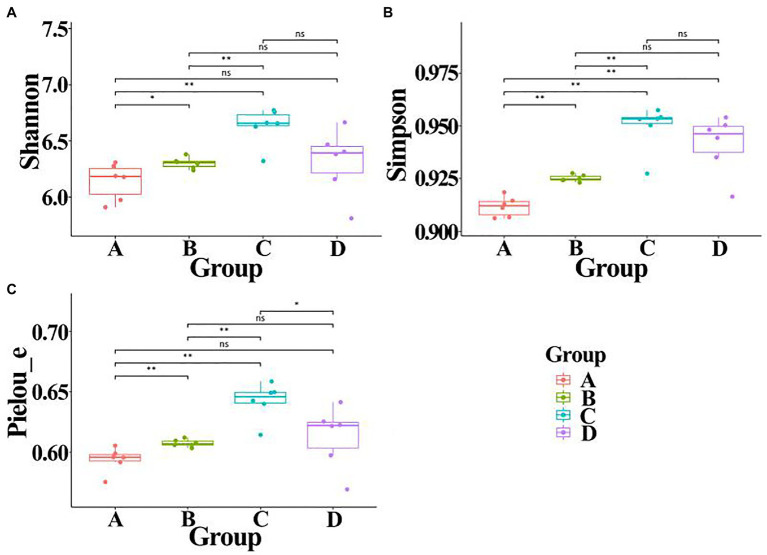
Alpha diversity of gut bacteria from healthy and ADHD children in the presence or absence of ZnONP. **(A)** Shannon index; **(B)** Simpson index; and **(C)** Pielou_e index. A: Healthy; B: healthy + ZnONP; C: ADHD; and D: ADHD + ZnONP. Vertical bars represent standard errors of the mean (*n* = 6). ^*^p ≤ 0.05; ^**^p ≤ 0.01. ns, not significant.

In order to calculate alpha diversity of gut bacteria, data of each sample were normalized to the same number of reads (33,680 sequences, the smallest number of sequences). A comprehensive analysis was carried out in this study by calculating Shannon, Simpson, and Pielou_e indexes. Result of Figure 6 indicated that the community diversity index of gut bacteria from ADHD children was significantly higher in the three different indexes than that of healthy children (*p* < 0.01; Figure 3). This is different with the result of Aarts et al. (2017), who found that there was no difference in alpha diversity of gut microbiota between patients with ADHD and controls using 16S rRNA marker gene sequencing. However, in agreement with the data of our study, Wang et al. (2020) found that the gut microbiota communities in ADHD patients showed a significantly higher Shannon index than the control subjects by using 16S rRNA V3V4 amplicon sequencing.

**Figure 7:**
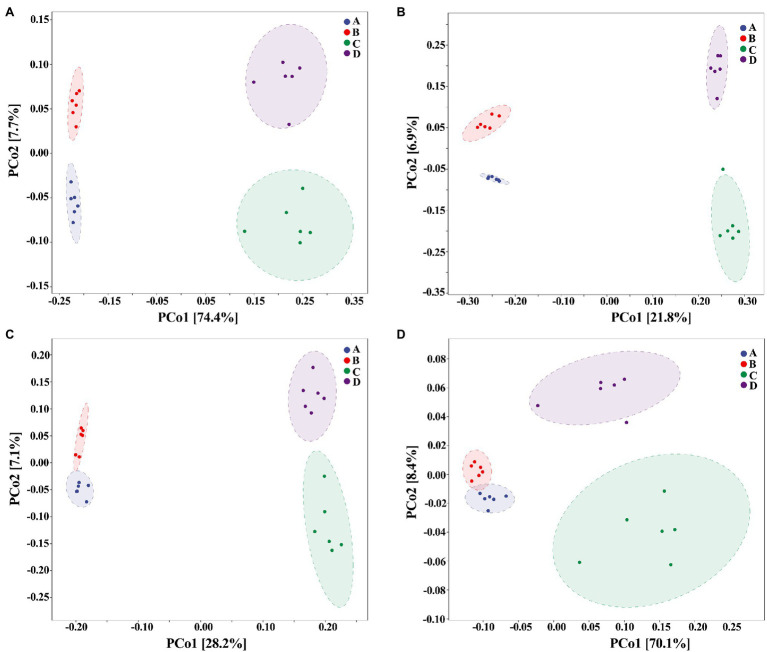
Beta diversity of gut bacteria by principal coordinates analysis (PCoA; *n* = 6) of **(A)** Bray–Curtis, **(B)** Jaccard, **(C)** unweighted UniFrac, and **(D)** weighted UniFrac. A: Healthy; B: healthy + ZnONP; C: ADHD; and D: ADHD + ZnONP.

On the other hand, our results also showed that the biosynthesized NPs had a differential effect between healthy and ADHD children in the community diversity index of gut bacteria. Indeed, the addition of ZnONP caused a significant (*p* < 0.05 or 0.01) increase in the three different community diversity index of gut bacteria from healthy children. In contrast, the Pielou_e index was significantly (*p* < 0.05) reduced, while Shannon and Simpson indexes of gut bacteria from ADHD children were unaffected by ZnONP (Figure 6). Thus, it can be inferred from this result that there was a difference in the community structure of gut bacteria between healthy and ADHD children. The beta diversity of gut bacteria in fecal samples from healthy and ADHD children in the presence or absence of ZnONP was further analyzed by using PCoA (principal coordinates analysis) of Bray–Curtis, Jaccard, unweighted UniFrac, and weighted UniFrac at the ASVS level. In general, PCoA analysis of the four different indicators showed a 3.5–74.4% variation of bacterial communities among these fecal samples. In detail, PC1 explained 74.4, 21.8, 28.2, and 70.1% of the variation, respectively, between healthy and ADHD children in Bray–Curtis, Jaccard, unweighted UniFrac, and weighted UniFrac parameters, while PC2 explained 7.7, 6.9, 7.1, and 3.5% of the variation, respectively, between with and without NPs in the above-mentioned four different indicators (Figure 7).

**Figure 8:**
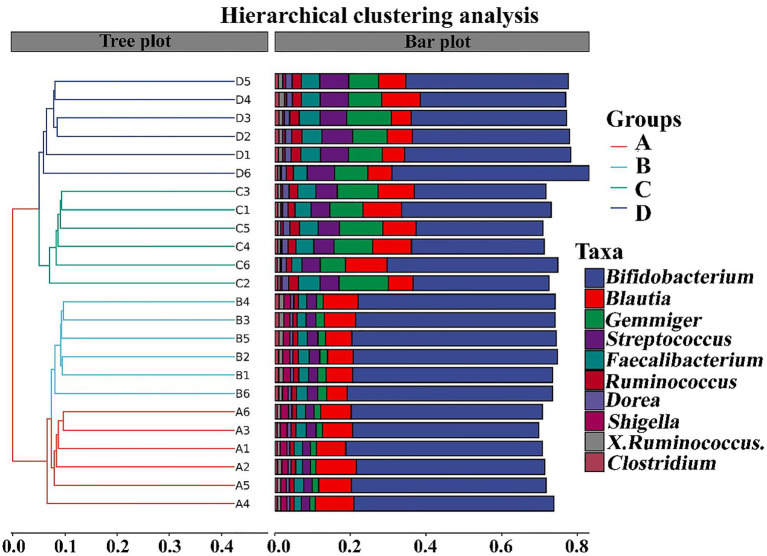
Analysis of hierarchical clustering constructed at the genus level by Bray–Curtis dissimilarity (*n* = 6). A: Healthy; B: healthy + ZnONP; C: ADHD; and D: ADHD + ZnONP.

In contrast with the result, Wang et al. (2020) found that the gut microbiomes were similar between the healthy controls and ADHD patients based on the unweighted UniFrac and weighted UniFrac plots. It can be inferred from these results that there was obvious differences in gut bacterial communities among the tested samples, which could be effectively differentiated by the above-mentioned four different indicators (Figure 7). However, the differential effect depends on the four selected parameters and the two principle components. Indeed, ADHD showed a greater differential effect than that of NP. In agreement with the result of diversity analysis, we also found that ADHD has a greater influence in change of gut bacterial communities than NPs based on the analysis of hierarchical clustering, which was constructed at the genus level by Bray–Curtis dissimilarity (Figure 8).

### Species Composition Determined by 16S rRNA Amplicon Sequencing

**Figure 9:**
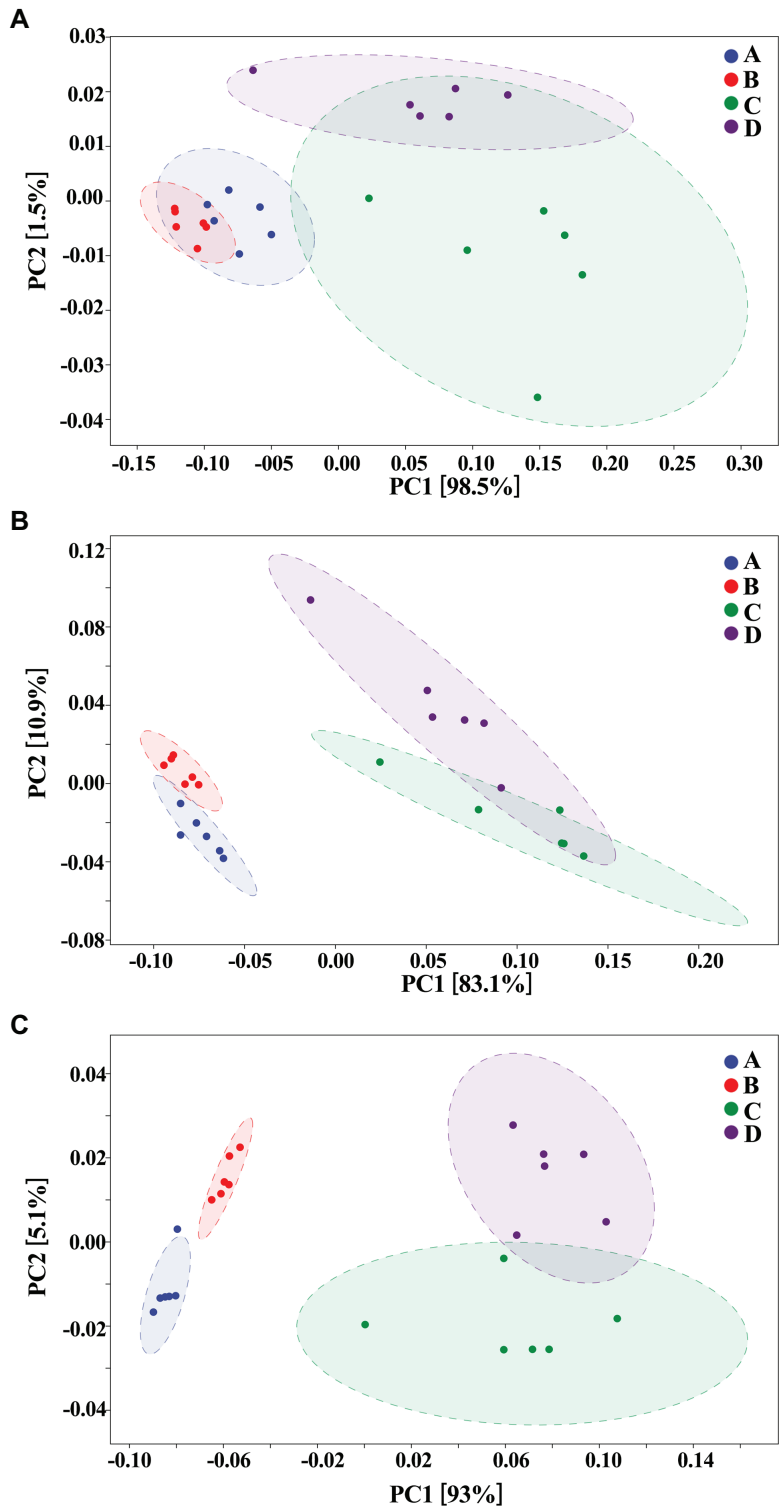
Principal components analysis (PCA; *n* = 6) at **(A)** phylum, **(B)** genus, and **(C)** species levels. A: Healthy; B: healthy + ZnONP; C: ADHD; and D: ADHD + ZnONP.

**Figure 10:**
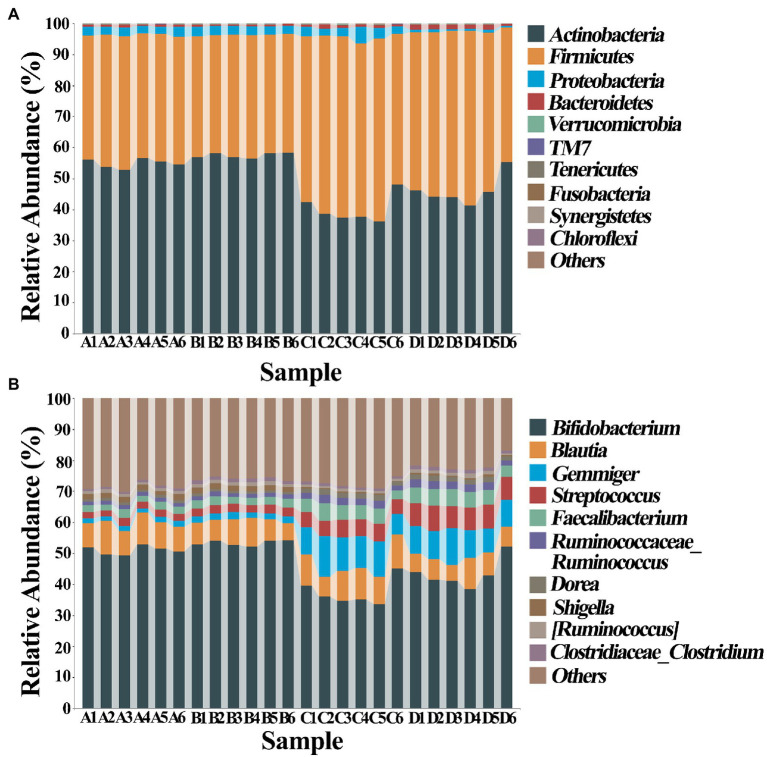
Classification and composition diagram (*n* = 6) at **(A)** phylum and **(B)** genus levels. A: Healthy; B: healthy + ZnONP; C: ADHD; and D: ADHD + ZnONP.

The composition of gut bacteria was determined from six replicates of each samples by PCA of phylum, genus, and species, which showed that there was an overall similarity in bacterial community structure within each sample. However, the community structure differed in different samples. Indeed, the result of PCA showed that there was a 95.6, 83.1, and 93% total variation between healthy and ADHD children in PC1 at the phylum, genus, and species levels, respectively, while there was a 3.5, 10.9, and 5.1% total variation between with and without NPs in PC2 at the phylum, genus, and species levels, respectively (Figure 9). This result consists of the data of diversity analysis, which indicated that ADHA achieved a greater change of gut bacterial communities than NPs. Following the comparison with the greengene database, we found that the top eight phylum of gut bacteria from these samples were *Actinobacteria*, *Firmicutes*, *Proteobacteria*, *Bacteroide*, *Verrucomicrobia*, TM7, *Tenericutes*, and *Fusobacteria*, of which *Actinobacteria* (49.6%) and *Firmicutes* (46.8%) were the main groups. At the genus level, there was no difference in the taxonomic composition between healthy children in presence and absence of NPs, as well as between ADHD children in presence and absence of NPs, but there was a difference between healthy and ADHD children. For example, the relative abundances of *Bifidobacterium* and *Gemmiger* in healthy children NPs were 52.1 and 9.7%, respectively; in contrast, the two genus in ADHD children were 40.3 and 1.8%, respectively, regardless of the presence or absence of NPs (Figure 10).

**Figure 11:**
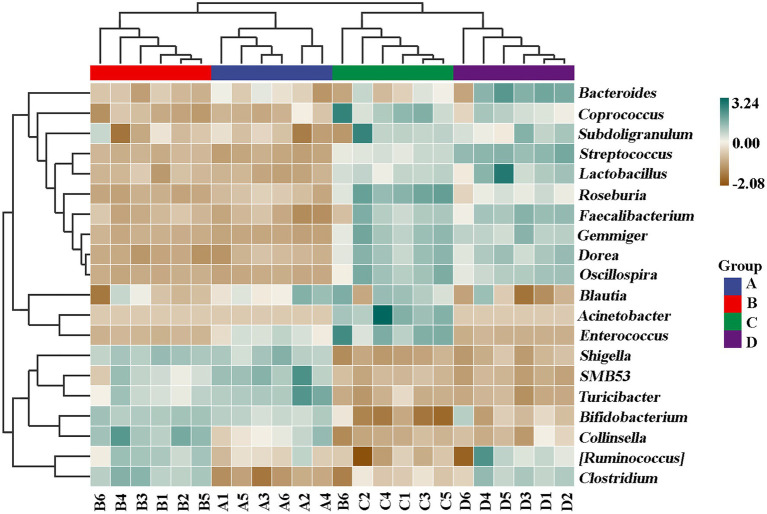
Composition and distribution of the main 20 genus among samples (*n* = 6).

In order to further compare the differences in species composition between the samples and display the trend of species abundance distribution of each sample, the heat map was draw by selecting the abundance data of the top 20 genera with average abundance. As shown in Figure 11, the results of this study revealed a significant change in the intestinal microbial community structure between healthy and ADHD children, which is in agreement with the data of previous reports (Stevens et al., 2019; Wang et al., 2020). Indeed, *Shigella*, *SMB53*, *Turicibacter*, *Shigella*, *Bifidobacterium*, *Collinsella*, *Ruminococcus*, and *Clostridium* are enriched in healthy children regardless of the presence or absence of NPs, while *Roseburia*, *Gemmiger*, *Acinetobacter*, *Enterococcus*, *Bacteroides*, *Streptococcus*, and *Faecalibacterium* were enriched in ADHD children regardless of the presence or absence of NPs. However, different bacterial species were changed because of Wang et al. (2020), who revealed that the relative abundance of *Bacteroides coprocola* was decreased, while the relative abundance of *Bacteroides uniformis*, *Bacteroides ovatus*, and *Sutterella stercoricanis* were increased in the ADHD group.

Undoubtedly, determination of ADHD-associated gut bacteria will open the possibility for new potential intervention strategies in the prevention of this disease; however, the conflicted results were often found in different studies in this field; for example, although the correlation of the genus *Bifidobacterium* with ADHD symptoms has been identified in several recent studies, the outcomes of *Bifidobacterium* in ADHD is still controversial. Several studies found that *Bifidobacterium* has a protective effect against ADHD, whereas Aarts et al. (2017) reported that ADHD patients had a 12.7–20.5 increased *Bifidobacterium* genus to controls. This may be mainly due to the difference in the objective and method of study. In general, this study indicated that ADHD caused the reduction in number of gut bacteria but increased the diversity of community, suggesting that the diversity of microbiome community may be related to the development of ADHD. Interestingly, the change of gut bacteria in ADHD is similar to that in ASD (autism spectrum disorder, another children disease) children in our previous studies (Yu et al., 2013, 2020). This can be justified by the result of many studies, which have found that comorbidity is high in ASD and ADHD individuals (Bölte et al., 2014). Fortunately, our results clearly indicated that NPs can decrease the diversity of microbiome community in ADHD children. Furthermore, some NP-resistant bacteria were successfully isolated, purified, and identified in this study. Therefore, it can be inferred that NPs and its resistant strains may potentially modulate and regulate the gut microbiome community of ADHD children.

## Conclusion

In this study, we have biologically synthesized and characterized the ZnONPs by using bacterial strain RTN1. Moreover, we investigated the impact of biogenic ZnONPs on the gut microbiota of healthy and ADHD children in China based on the traditional plate method and amplicon sequencing analysis for the first time. Compared to healthy children, significant changes were noted in the number and composition of gut bacteria from the fecal samples of children with ADHD, indicating these bacteria residing in the gut may contribute to ADHD development. In addition, we found that NPs have a differential effect in composition of gut bacteria from healthy and ADHD children, while the NP-resistant strains in healthy children are also different from that in ADHD children. Overall, the result not only revealed the potential correlation of ADHD with gut bacteria but also found that biogenic ZnONPs could change the number and composition of gut bacteria. In particular, some gut bacteria that are resistant to biogenic ZnONPs have been successfully isolated and identified, which gave a new strategy to prevent ADHD by the combination of NP and its resistant bacteria. However, future studies are also needed to assess the effect of ZnONPs on the normal cells and beneficial microbiota, as well as animal trials for their large-scale applications.

## Data Availability Statement

The datasets presented in this study can be found in online repositories. The names of the repository/repositories and accession number(s) can be found at: https://www.ncbi.nlm.nih.gov/, MW664943, MW664944, MW664945, MW664946, MW664947, MW664948, and MW664949.

## Author Contributions

GZ, RY, and BL: conceptualization. GZ, RY, HJ, MZ, LL, TA, and BL: methodology and writing – original draft. RY, HJ, LL, and TA: software. GZ, FA, KA, and RY: data investigation. BL: supervision. KA, GZ, RY, and BL: visualization. GZ, RY, TA, HJ, MZ, LL, FA, KA, and BL: writing – review and editing. All authors contributed to the article and approved the submitted version.

## Conflict of Interest

The authors declare that the research was conducted in the absence of any commercial or financial relationships that could be construed as a potential conflict of interest.

## Publisher’s Note

All claims expressed in this article are solely those of the authors and do not necessarily represent those of their affiliated organizations, or those of the publisher, the editors and the reviewers. Any product that may be evaluated in this article, or claim that may be made by its manufacturer, is not guaranteed or endorsed by the publisher.

## References

[ref1] Aarts E. Ederveen T. H. Naaijen J. Zwiers M. P. Boekhorst J. Timmerman H. M.. (2017). Gut microbiome in ADHD and its relation to neural reward anticipation. PLoS One 12:e0183509. 10.1371/journal.pone.0183509, PMID: 28863139 PMC5581161

[ref2] Abid M. A. Kadhim D. A. Aziz W. J. (2020). Iron oxide nanoparticle synthesis using trigonella and tomato extracts and their antibacterial activity. Mater. Technol. 25, 1–8. 10.1080/10667857.2020.1863572

[ref3] Agarwal H. Menon S. Kumar S. V. Rajeshkumar S. (2018). Mechanistic study on antibacterial action of zinc oxide nanoparticles synthesized using green route. Chem. Biol. Interact. 286, 60–70. 10.1016/j.cbi.2018.03.008, PMID: 29551637

[ref4] Ahmed T. Noman M. Luo J. Muhammad S. Shahid M. Ali M. A.. (2021a). Bioengineered chitosan-magnesium nanocomposite: a novel agricultural antimicrobial agent against *Acidovorax oryzae* and *Rhizoctonia solani* for sustainable rice production. Int. J. Biol. Macromol. 168, 834–845. 10.1016/j.ijbiomac.2020.11.148, PMID: 33242551

[ref5] Ahmed T. Noman M. Shahid M. Shahid M. S. Li B. (2021b). Antibacterial potential of green magnesium oxide nanoparticles against rice pathogen *Acidovorax oryzae*. Mater. Lett. 282:128839. 10.1016/j.matlet.2020.128839

[ref6] Ahmed T. Shahid M. Noman M. Niazi M. B. K. Zubair M. Almatroudi A.. (2020). Bioprospecting a native silver-resistant *Bacillus safensis* strain for green synthesis and subsequent antibacterial and anticancer activities of silver nanoparticles. J. Adv. Res. 24, 475–483. 10.1016/j.jare.2020.05.011, PMID: 32566283 PMC7296185

[ref7] Ahmed T. Wu Z. Jiang H. Luo J. Noman M. Shahid M.. (2021c). Bioinspired green synthesis of zinc oxide nanoparticles from a native *Bacillus cereus* strain RNT6: characterization and antibacterial activity against rice panicle blight pathogens *Burkholderia glumae* and *B. gladioli*. Nanomaterials 11:884. 10.3390/nano11040884, PMID: 33808470 PMC8065826

[ref8] Ali M. Ahmed T. Wu W. Hossain A. Hafeez R. Islam Masum M.. (2020). Advancements in plant and microbe-based synthesis of metallic nanoparticles and their antimicrobial activity against plant pathogens. Nanomaterials 10:1146. 10.3390/nano10061146, PMID: 32545239 PMC7353409

[ref9] Alphandéry E. (2020). Bio-synthesized iron oxide nanoparticles for cancer treatment. Int. J. Pharm. 586:119472. 10.1016/j.ijpharm.2020.119472, PMID: 32590095

[ref10] Bölte S. Willfors C. Berggren S. Norberg J. Poltrago L. Mevel K.. (2014). The roots of autism and ADHD twin study in Sweden (RATSS). Twin Res. Hum. Genet. 17, 164–176. 10.1017/thg.2014.12, PMID: 24735654

[ref11] Boonchooduang N. Louthrenoo O. Chattipakorn N. Chattipakorn S. C. (2020). Possible links between gut–microbiota and attention-deficit/hyperactivity disorders in children and adolescents. Eur. J. Nutr. 59, 3391–3403. 10.1007/s00394-020-02383-1, PMID: 32918136

[ref12] Bundgaard-Nielsen C. Knudsen J. Leutscher P. D. Lauritsen M. B. Nyegaard M. Hagstrøm S.. (2020). Gut microbiota profiles of autism spectrum disorder and attention deficit/hyperactivity disorder: a systematic literature review. Gut Microbes 11, 1172–1187. 10.1080/19490976.2020.1748258, PMID: 32329656 PMC7524304

[ref13] Callahan B. J. McMurdie P. J. Rosen M. J. Han A. W. Johnson A. J. A. Holmes S. P. (2016). DADA2: high-resolution sample inference from illumina amplicon data. Nat. Methods 13, 581–583. 10.1038/nmeth.3869, PMID: 27214047 PMC4927377

[ref14] Checa-Ros A. Jeréz-Calero A. Molina-Carballo A. Campoy C. Muñoz-Hoyos A. (2021). Current evidence on the role of the gut microbiome in ADHD pathophysiology and therapeutic implications. Nutrients 13:249. 10.3390/nu13010249, PMID: 33467150 PMC7830868

[ref15] De Vadder F. Kovatcheva-Datchary P. Goncalves D. Vinera J. Zitoun C. Duchampt A.. (2014). Microbiota-generated metabolites promote metabolic benefits via gut-brain neural circuits. Cell 156, 84–96. 10.1016/j.cell.2013.12.016, PMID: 24412651

[ref16] Gericke M. Pinches A. (2006). Biological synthesis of metal nanoparticles. Hydrometallurgy 83, 132–140. 10.1016/j.hydromet.2006.03.019

[ref17] Hayashi W. Iwanami A. (2018). Biological mechanisms of ADHD. Brain Nerve 70, 1265–1277. 10.11477/mf.1416201172, PMID: 30416120

[ref18] Hiergeist A. Gessner J. Gessner A. (2020). Current limitations for the assessment of the role of the gut microbiome for attention deficit hyperactivity disorder (ADHD). Front. Psychiatry 11:623. 10.3389/fpsyt.2020.00623, PMID: 32670122 PMC7332545

[ref19] Karmakar A. Goswami R. Saha T. Maitra S. Roychowdhury A. Panda C. K.. (2017). Pilot study indicate role of preferentially transmitted monoamine oxidase gene variants in behavioral problems of male ADHD probands. BMC Med. Genet. 18:109. 10.1186/s12881-017-0469-5, PMID: 28982350 PMC5629801

[ref20] Kummara S. Patil M. B. Uriah T. (2016). Synthesis, characterization, biocompatible and anticancer activity of green and chemically synthesized silver nanoparticles–a comparative study. Biomed. Pharmacother. 84, 10–21. 10.1016/j.biopha.2016.09.003, PMID: 27621034

[ref21] Li B. Wang X. Chen R. Huangfu W. Xie G. (2008). Antibacterial activity of chitosan solution against Xanthomonas pathogenic bacteria isolated from *Euphorbia pulcherrima*. Carbohydr. Polym. 72, 287–292. 10.1016/j.carbpol.2007.08.012

[ref22] Luo Y. Weibman D. Halperin J. M. Li X. (2019). A review of heterogeneity in attention deficit/hyperactivity disorder (ADHD). Front. Hum. Neurosci. 13:42. 10.3389/fnhum.2019.00042, PMID: 30804772 PMC6378275

[ref23] Malaikozhundan B. Vaseeharan B. Vijayakumar S. Thangaraj M. P. (2017). *Bacillus thuringiensis* coated zinc oxide nanoparticle and its biopesticidal effects on the pulse beetle, *Callosobruchus maculatus*. J. Photochem. Photobiol. B 174, 306–314. 10.1016/j.jphotobiol.2017.08.014, PMID: 28818776

[ref24] Martins-Silva T. Salatino-Oliveira A. Genro J. P. Meyer F. D. Li Y. Rohde L. A.. (2021). Host genetics influences the relationship between the gut microbiome and psychiatric disorders. Prog. Neuro Psychopharmacol. Biol. Psychiatry 106:110153. 10.1016/j.pnpbp.2020.110153, PMID: 33130294

[ref25] Masum M. Islam M. Siddiqa M. Ali K. A. Zhang Y. Abdallah Y.. (2019). Biogenic synthesis of silver nanoparticles using *Phyllanthus emblica* fruit extract and its inhibitory action against the pathogen *Acidovorax oryzae* strain RS-2 of rice bacterial brown stripe. Front. Microbiol. 10:820. 10.3389/fmicb.2019.00820, PMID: 31110495 PMC6501729

[ref26] Ogunyemi S. O. Abdallah Y. Zhang M. Fouad H. Hong X. Ibrahim E.. (2019a). Green synthesis of zinc oxide nanoparticles using different plant extracts and their antibacterial activity against *Xanthomonas oryzae* pv. *oryzae*. Artif. Cells Nanomed. Biotechnol. 47, 341–352. 10.1080/21691401.2018.1557671, PMID: 30691311

[ref27] Ogunyemi S. O. Zhang M. Abdallah Y. Ahmed T. Qiu W. Ali M.. (2020). The bio-synthesis of three metal oxide nanoparticles (ZnO, MnO_2_, and MgO) and their antibacterial activity against the bacterial leaf blight pathogen. Front. Microbiol. 11:588326. 10.3389/fmicb.2020.588326, PMID: 33343527 PMC7746657

[ref28] Ogunyemi S. O. Zhang F. Abdallah Y. Zhang M. Wang Y. Sun G.. (2019b). Biosynthesis and characterization of magnesium oxide and manganese dioxide nanoparticles using *Matricaria chamomilla* L. extract and its inhibitory effect on *Acidovorax oryzae* strain RS-2. Artif. Cells Nanomed. Biotechnol. 47, 2230–2239. 10.1080/21691401.2019.1622552, PMID: 31161806

[ref29] Pietroiusti A. Magrini A. Campagnolo L. (2016). New frontiers in nanotoxicology: gut microbiota/microbiome-mediated effects of engineered nanomaterials. Toxicol. Appl. Pharmacol. 299, 90–95. 10.1016/j.taap.2015.12.017, PMID: 26723910

[ref30] Sampson T. R. Mazmanian S. K. (2015). Control of brain development, function, and behavior by the microbiome. Cell Host Microbe 17, 565–576. 10.1016/j.chom.2015.04.011, PMID: 25974299 PMC4442490

[ref31] Saravanan M. Gopinath V. Chaurasia M. K. Syed A. Ameen F. Purushothaman N. (2018). Green synthesis of anisotropic zinc oxide nanoparticles with antibacterial and cytofriendly properties. Microb. Pathog. 115, 57–63. 10.1016/j.micpath.2017.12.039, PMID: 29248514

[ref32] Selvarajan E. Mohanasrinivasan V. (2013). Biosynthesis and characterization of ZnO nanoparticles using *Lactobacillus plantarum* VITES07. Mater. Lett. 112, 180–182. 10.1016/j.matlet.2013.09.020

[ref33] Stevens A. J. Purcell R. V. Darling K. A. Eggleston M. J. Kennedy M. A. Rucklidge J. J. (2019). Human gut microbiome changes during a 10 week randomised control trial for micronutrient supplementation in children with attention deficit hyperactivity disorder. Sci. Rep. 9:10128. 10.1038/s41598-019-46146-3, PMID: 31300667 PMC6625977

[ref34] Suresh J. Pradheesh G. Alexramani V. Sundrarajan M. Hong S. I. (2018). Green synthesis and characterization of zinc oxide nanoparticle using insulin plant (*Costus pictus* D. *Don*) and investigation of its antimicrobial as well as anticancer activities. Adv. Nat. Sci. Nanosci. Nanotechnol. 9:15008. 10.1088/2043-6254/aaa6f1

[ref35] Szopinska-Tokov J. Dam S. Naaijen J. Konstanti P. Rommelse N. Belzer C.. (2020). Investigating the gut microbiota composition of individuals with attention-deficit/hyperactivity disorder and association with symptoms. Microorganisms 8:406. 10.3390/microorganisms8030406, PMID: 32183143 PMC7143990

[ref36] Wan L. Ge W.-R. Zhang S. Sun Y.-L. Wang B. Yang G. (2020). Case-control study of the effects of gut microbiota composition on neurotransmitter metabolic pathways in children with attention deficit hyperactivity disorder. Front. Neurosci. 14:127. 10.3389/fnins.2020.00127, PMID: 32132899 PMC7040164

[ref37] Wang L.-J. Yang C.-Y. Chou W.-J. Lee M.-J. Chou M.-C. Kuo H.-C.. (2020). Gut microbiota and dietary patterns in children with attention-deficit/hyperactivity disorder. Eur. Child Adolesc. Psychiatry 29, 287–297. 10.1007/s00787-019-01352-2, PMID: 31119393

[ref38] Yu R. Wu Z. Wang S. Zhang M. Zhou G. Li B. (2020). Isolation, identification and characterization of propionic acid bacteria associated with autistic spectrum disorder. Microb. Pathog. 147:104371. 10.1016/j.micpath.2020.104371, PMID: 32634613

[ref39] Yu R.-R. Xu Y. Wang Y.-G. Qiu F.-Y. (2013). Chemical environment influence the incidence of childhood autism. Asian J. Chem. 25, 8835–8837. 10.14233/ajchem.2013.14870

